# CRISPR/Cas9 small promoter deletion in *H19* lncRNA is associated with altered cell morphology and proliferation

**DOI:** 10.1038/s41598-021-97058-0

**Published:** 2021-09-15

**Authors:** Renan da Silva Santos, Daniel Pascoalino Pinheiro, Louhanna Pinheiro Rodrigues Teixeira, Sarah Leyenne Alves Sales, Maria Claudia dos Santos Luciano, Mayara Magna de Lima Melo, Ronald Feitosa Pinheiro, Kaio César Simiano Tavares, Gilvan Pessoa Furtado, Claudia Pessoa, Cristiana Libardi Miranda Furtado

**Affiliations:** 1 Department of Physiology and Pharmacology, Drug Research and Development Center, Federal University of Ceará, Fortaleza, Brazil; 2 Experimental Biology Nucleus, University of Fortaleza, Fortaleza, Ceará Brazil; 3 Department of Clinical Medicine, Drug Research and Development Center, Federal University of Ceará, Fortaleza, Brazil; 4 Sector of Biotechnology, Oswaldo Cruz Foundation, FIOCRUZ-Ceará, Fortaleza, Brazil; 5 Drug Research and Development Center, Postgraduate Program in Translational Medicine, Federal University of Ceará, Fortaleza, Brazil; 6 Department of Gynecology and Obstetrics, Ribeirao Preto Medical School, University of São Paulo, Ribeirão Prêto, SP Brazil

**Keywords:** Cancer epigenetics, Mechanisms of disease

## Abstract

The imprinted *H19* long non-coding RNA, a knowing oncofetal gene, presents a controversial role during the carcinogenesis process since its tumor suppressor or oncogenic activity is not completely elucidated. Since *H19* lncRNA is involved in many biological pathways related to tumorigenesis, we sought to develop a non-cancer lineage with CRISPR-Cas9-mediated *H19* knockdown (*H19*-) and observe the changes in a cellular context. To edit the promoter region of *H19*, two RNA guides were designed, and the murine C2C12 myoblast cells were transfected. *H19* deletion was determined by DNA sequencing and gene expression by qPCR. We observed a small deletion (~ 60 bp) in the promoter region that presented four predicted transcription binding sites. The deletion reduced *H19* expression (30%) and resulted in increased proliferative activity, altered morphological patterns including cell size and intracellular granularity, without changes in viability. The increased proliferation rate in the *H19*- cell seems to facilitate chromosomal abnormalities. The *H19*- myoblast presented characteristics similar to cancer cells, therefore the *H19* lncRNA may be an important gene during the initiation of the tumorigenic process. Due to CRISPR/Cas9 permanent edition, the C2C12 *H19*- knockdown cells allows functional studies of *H19* roles in tumorigenesis, prognosis, metastases, as well as drug resistance and targeted therapy.

## Introduction

The eutherian mammalian genome presents a subset of genes with monoallelic expression in a parent-of-origin manner, in which only the maternal or paternal allele will be functionally active^1^. The first imprinted genes were identified in 1991 and since then 121 imprinted genes have been mapped in humans and 136 in mice^1,2^. The *H19* long non-coding gene is probably one of the most studied imprinted genes^3^ and is associated with *IGF2* (Insulin-Like Growth Factor 2) as a cluster of regulation. *H19* and *IGF2* activity is regulated by an imprinting control region (ICR) that is a differentially methylated region (DMR) located upstream of H19 called H19DMR or ICR1^4^.

**Figure 1:**
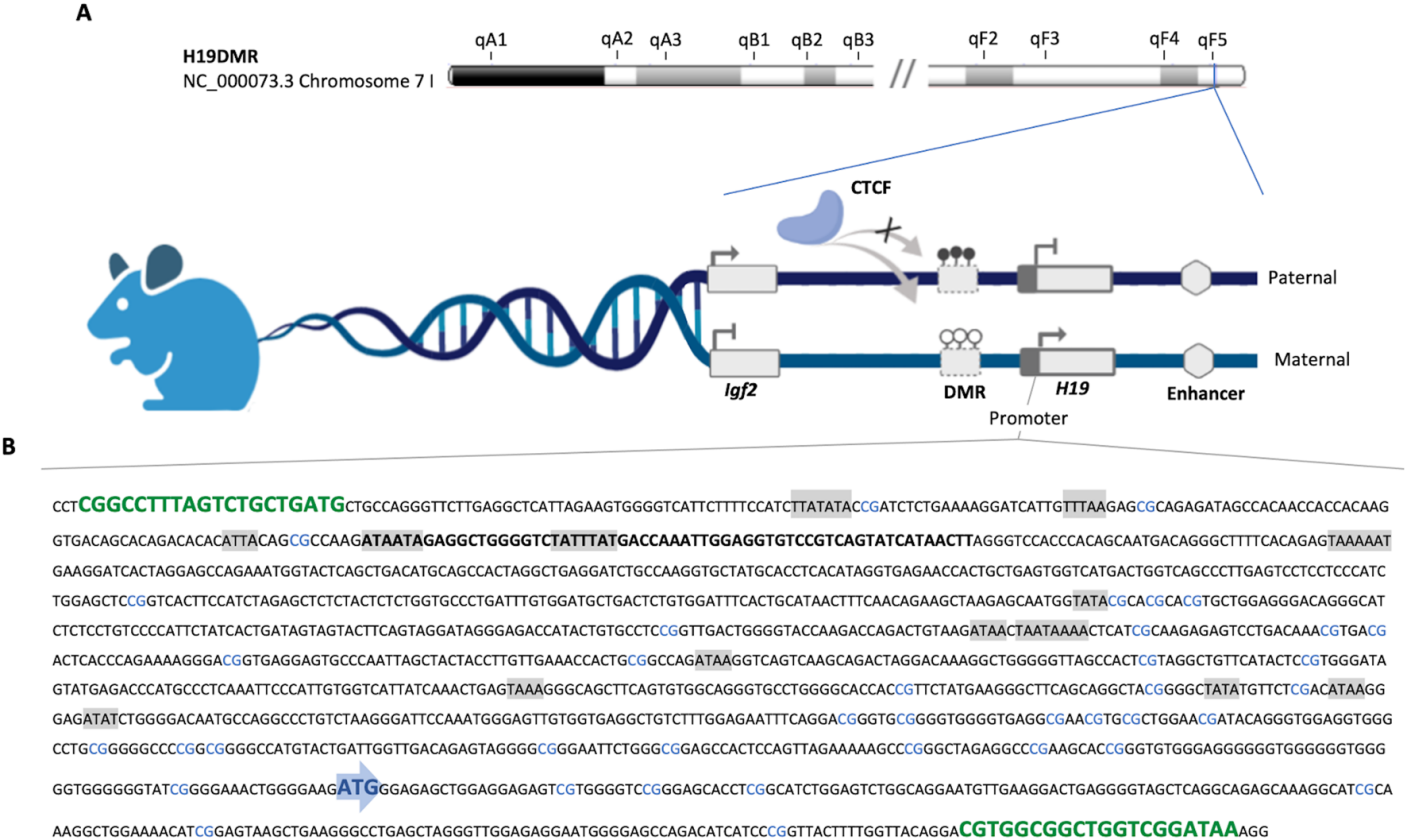
(**A**) Schematic representation of murine H19DMR and *H19* promoter sequence (NC_000073.3 Chromosome 7 F5). The H19DMR is an imprinting control region that regulates *H19* and *IGF2* gene activity in an enhancer competition and insulator binding model. The differentially methylated region (DMR) near to *H19* promoter is unmethylated in the maternal allele, which allows the attachment of the CTCF insulator protein and *H19* expression. The insulator activity modifies the chromatin and prevents the enhancer activation of the *IGF2*, which is silenced. On the paternal allele, the methylation blocks the insulator biding, whereas makes conformational modifications in the *H19* promoter, which is silenced on the paternal allele, while the enhancer acts on the *IGF2* gene that is paternally expressed. (**B**) *H19* predicted promoter region sequence (GRCm38/mm10 Chr7:142577859-142579404). The guide RNAs (gRNAs) (green) were designed to flank the promoter region, including 1301 base of pairs (bp) upstream and 225 bp downstream to the start codon “ATG” (blue arrow) of the first exon. 14 TATA Box sequences (highlighted in grey) were identified.

The H19DMR contains a CTCF-binding domain (CCCTC-binding factor) with an insulator activity and enhancer competition model of gene regulation. Under normal conditions, the methylation on the paternal allele blocks the binding of the insulator protein and makes conformational modifications in the *H19* promoter, which is silenced, while the enhancer acts on the *IGF2* gene that is paternally expressed (Fig. 1). In the maternal allele, the absence of methylation (unmethylated) allows the attachment of the insulator protein that blocks the enhancer activation of the *IGF2,* which is silenced, thereby acting in the *H19* promoter, which is maternally expressed^4^. The complex process of *H19* transcription results in a long non-coding RNA (lncRNA) and two conserved microRNAs, miR-675-3p and miR-675-5p, that attend to regulate the expression of important genes such as *IGF1R*, *CADHERIN-13, CADHERIN-11, RUNX1, RB* and *TP53*^5^.

The monoallelic expression of *H19* is essential to important biological processes and biallelic expression or loss of imprinting (LOI) in the H19DMR is related to several diseases including congenital disorders, neurodevelopmental alterations, and cancer^6,7^. The three non-coding RNAs transcribed by *H19* can interact across multiple pathways, thus the role of *H19* in tumor development is not completely elucidated and it is speculated to act as a tumor suppressor or oncogene^8^. Aberrant *H19* expression pattern, as increased and reduced expression levels, has already been observed in more than 30 types of tumors, such as Wilms tumorigenesis and rhabdosarcoma in newborns, suggesting an important role in tumorigenesis^8,9^. In addition, the *H19* knockdown, in hypoxia conditions, was found to be related to decreased expression of genes involved in different pathways, as angiogenesis (*ANG*), anti-apoptotic activity (*AKT1*), signal transduction for cell cycle (*RASSF2*), protein transport (*RAB4A*) and other events^9^.

*H19* knockout cell lines have been developed to understand the main targets of its transcripts, however, most models are transient, using shRNA (Short Hairpin RNA) or siRNA (Short Interfering RNA)^10,11^. Besides the absence of stability in these gene-silencing tools, those manipulated cell lines may suffer compensatory effects by increasing or reducing the expression of the target transcript in response to RNAi (RNA interference)^12^. CRISPR/Cas9 (Clustered Regularly Interspaced Short Palindromic Repeats) differs from other gene-editing or RNAi platforms as it directly targets the DNA strand through hybridization of a guide RNA (gRNA). The restrictions for a gRNA to find a target in the eukaryotic genome are small, which allows it to achieve specific modifications^13^.

In the context of studies for loss of function (LOF), CRISPR/Cas9 is considered one of the most robust and consistent tools for generating stable knockouts and knockdowns due to the permanent damage generated in the DNA and the reduced cytotoxic effect^13,14^. To clarify the role of a gene in a biological process, one strategy is to disturb the expression by genome editing and observe the subsequent cellular alterations^15^. Since the *H19* has important roles in many biological pathways such as cell growth and development, and its mechanisms involved in tumorigenic processes are not completely elucidated, we sought to develop a non-cancer cell line with a permanent knockdown of the *H19* gene by CRISPR/Cas9 and evaluate the results in gene expression and cell growth.

## Results

### CRISPR/Cas9-mediated H19 edition reduces gene expression

**Figure 2:**
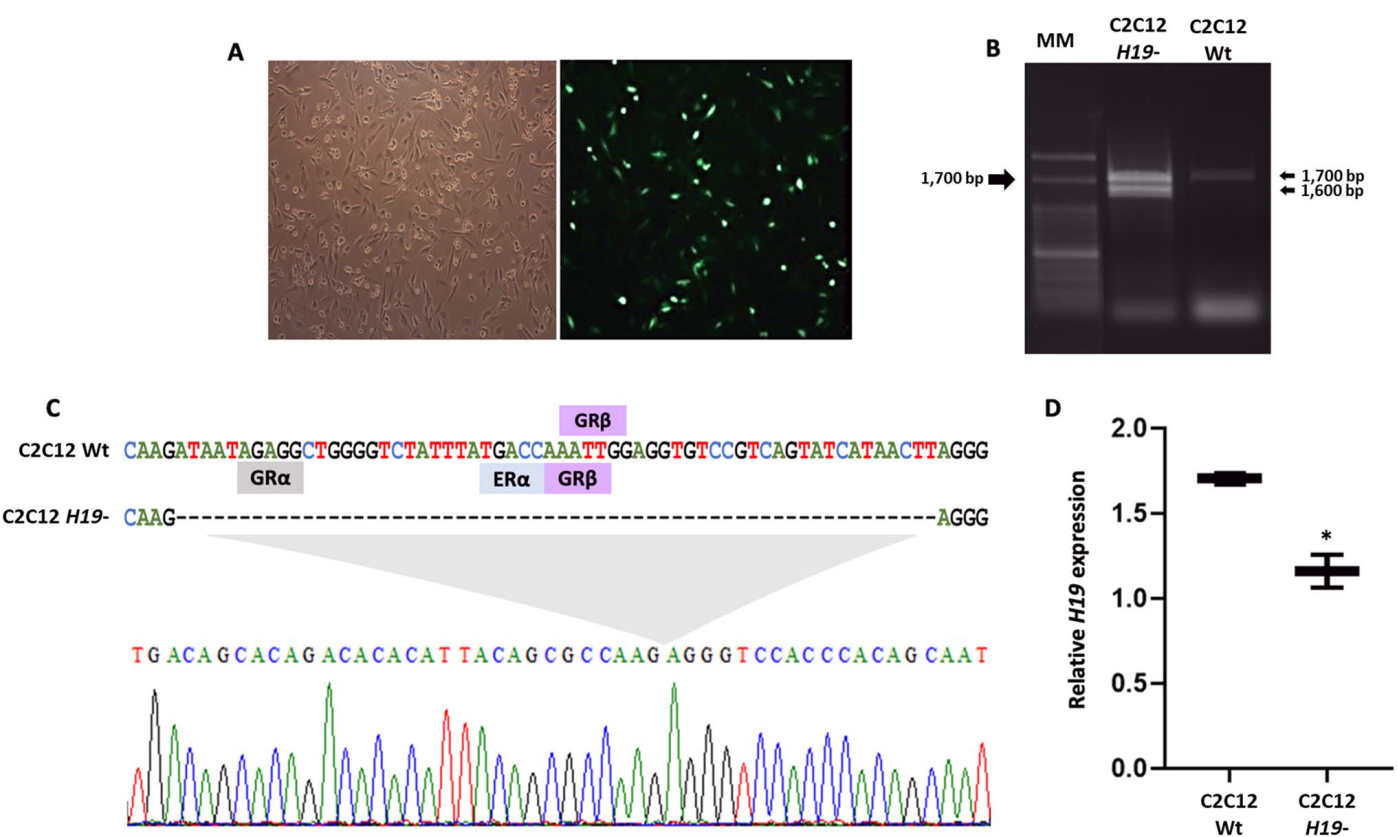
CRISPR/Cas9 screening and molecular characterization. (**A**) Transfection confirmation by GFP detection in C2C12 myoblast cells (100X magnification). (**B**) Amplification of the promoter region of C2C12 Wt and C2C12 *H19*- after CRISPR-Cas9 edition (1686 bp). MM, Molecular Marker (100 bp). (**C**) Alignment between *H19* reference and C2C12 *H19-* colony sequence. The chromatogram shows the location of the deleted sequence. Transcription factors with binding sites in the sequence were predicted by the PROMO bioinformatics. (**D**) Relative expression of C2C12 Wt and C2C12 *H19*- colonies for *H19* by qPCR (*p = 0.01).

The guide RNA (gRNA) construction for gene edition was designed to flank the promoter region of murine *H19* lncRNA, predicted by the presence of TATA-box elements and transcriptional initiation sites. The region included 14 TATA-box sequences upstream of the start codon “ATG” of the first exon (Fig. 1B). To optimize the deletion through double-stranded breaks and decrease in *H19* gene expression, the gRNA1 was designed to cleavage 1301 base pairs (bp) upstream to the methionine start codon (AUG) and the gRNA2 to cleave 225 nucleotides downstream the initiation codon. pX458 plasmids containing both gRNAs were co-transfected in C2C12 myoblast, which was confirmed by the green fluorescent protein (GFP) expression (Fig. 2A). Single-cell isolation by limiting dilution resulted in a total of 76 colonies, and after amplification 75 showed a PCR product around 1686 bp corresponding to the promoter region without deletion. Only one colony presented two PCR products, one similar to the non-edited cell and a smaller amplicon with deletion of about 100 bp (Fig. 2B).

The unexpected edition was confirmed by DNA sequencing that showed a deletion of 60 nucleotides in the promoter region of the *H19* gene, 146 bp downstream from the gRNA1. According to in silico prediction of transcription factor binding sites (TFBS), the promoter region deleted presented regulatory elements, including a loss of four potential functional binding regions (Fig. 2C). Among the identified TFBS, three belong to the glucocorticoid receptor (GR) family, including one alpha (GR-alpha) and two beta (GR-beta) subunits, and one corresponds to estrogen receptor alpha (ER-alpha). The deletion in the promoter region, even though it is smaller than expected, reduced *H19* expression in 1.47-folds in the C2C12 *H19-* knockdown cell when compared to the wild-type colony, C2C12 Wt (*p* = 0.01) (Fig. 2D). Therefore, the reduced gene expression and loss of TFBS may contribute to cellular alterations in the edited C2C12 *H19*-.

### H19 knockdown promotes cellular growth, morphological changes, and chromosomal alterations

**Figure 3:**
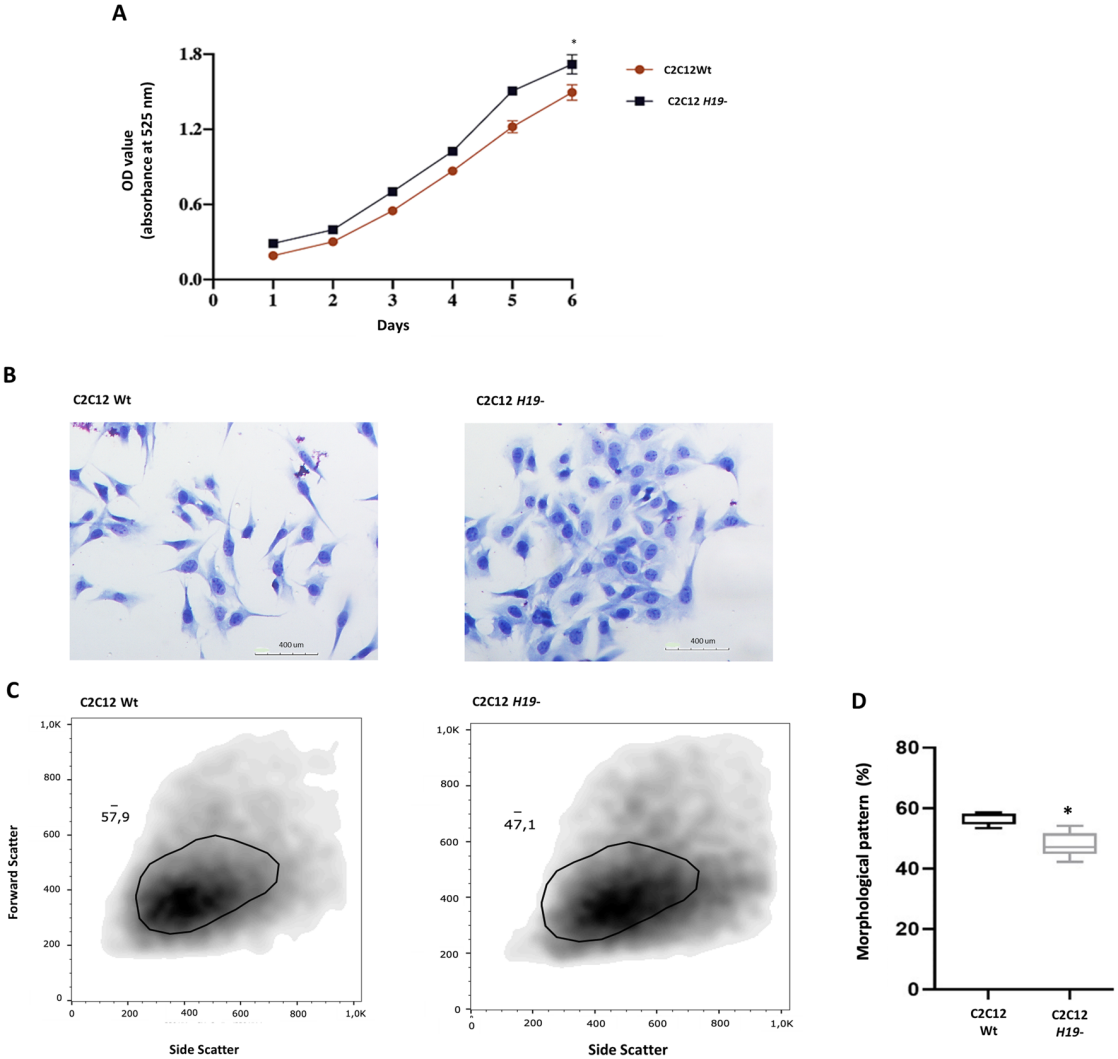
Cell proliferation assay and morphology. (**A**) MTT cell proliferation assay during six days of culture showing increased proliferation rate in *H19* knockdown (*H19*-) cells (*p = 0.0025). (**B**) Myoblastic cell morphology was not altered in both C2C12 wild-type (Wt) and *H19-* cells and an increased number of cells in the field were observed (100 × Magnification). (**C**) Flow cytometry histograms showing increased cell size (forward scatter, FS) and cytoplasmatic granularity (side scatter, SS) in the H19- cells. (**D**) Overlay of the FS and SS showing the percentage of altered morphological patterns in C2C12 *H19*- cells (*p = 0.001).

The *H19* knockdown in mouse myoblast cells resulted in an increased cell growth curve (*p* = 0.0025) (Fig. 3A). The modified Romanowsky staining for morphological analysis of both C2C12 *H19*- and C2C12 Wt cells suggests that C2C12 *H19*- preserves the standard myoblast characteristics (Fig. 3B). However, a higher number of cells in the fields captured was observed in the C2C12 *H19*- group, which corroborates with the increased proliferative activity. Analysis of cell morphology by flow cytometry showed altered internal complexity (*p* = 0.0001) (Fig. 3D), such as increased cell size (Foward Scater) and intracellular granularity (Side Scater) in C2C12 *H19-* cells (Fig. 3C).

**Figure 4:**
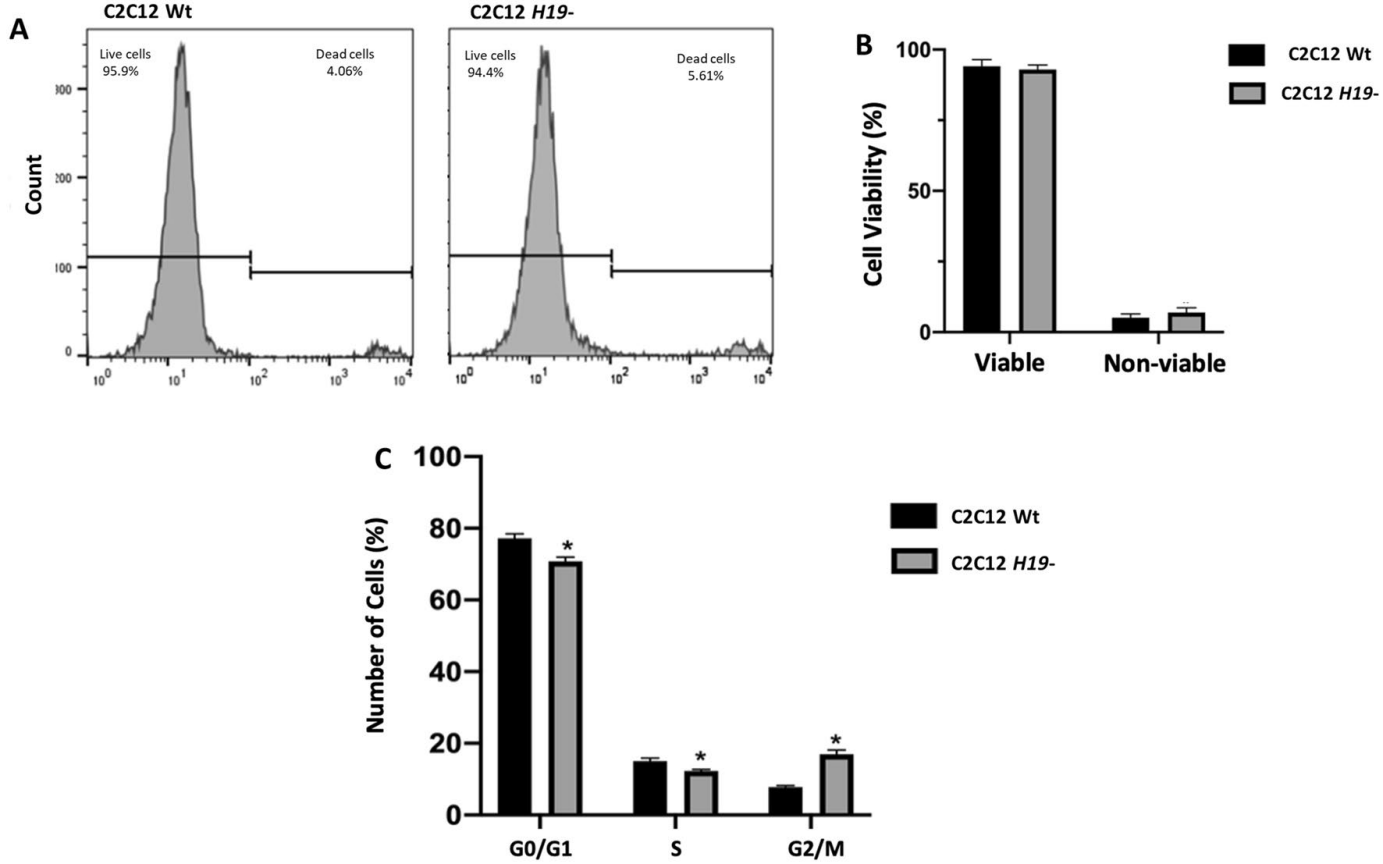
Flow cytometry viability and cell cycle analysis. Cell viability was not altered in the C2C12 *H19-* cells; (**A**) Graphical representation and (**B**) Percentage of viable and non-viable cells in Wt and H19- cells. (**C**) Cell cycle was altered in the H19- knockdown cells with a reduced percentage of cells in G0/G1 (*p* = 0.0003) and S (*p* =  0.001) phases and increased percentage of cells in G2/M (*p* < 0.0001).

**Figure 5:**
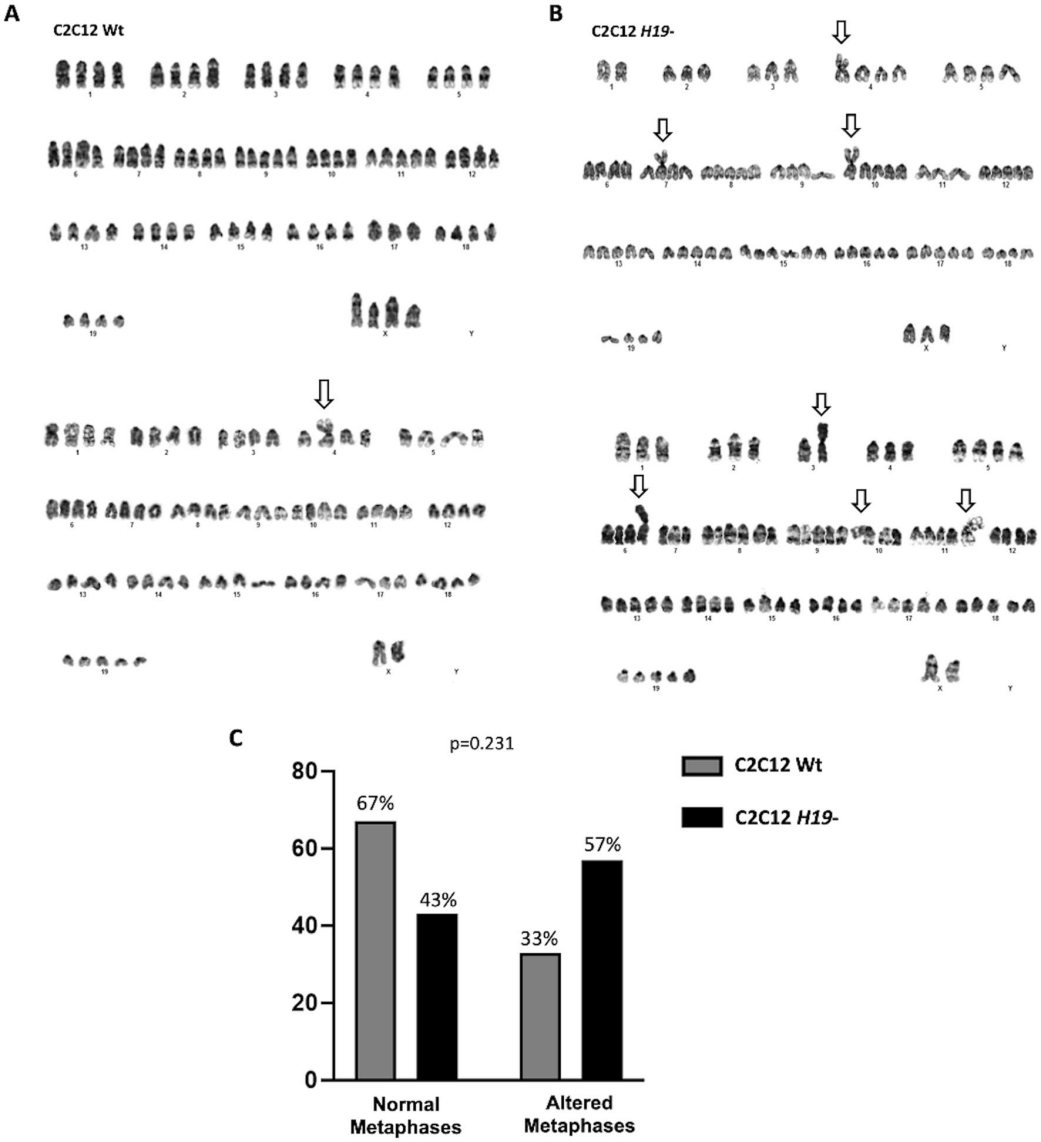
Karyotype analysis. C2C12 Wt (**A**) and C2C12 *H19-* colonies (**B**) showing chromosomal alterations, including increased translocations (arrow) in the knockdown cells. (**C**) Normal and altered metaphases in the C2C12 Wt and H19- cells (*p* = 0.0231).

The increased cell growth and internal complexity alterations seem not to be related to alterations in cellular viability in the C2C12 *H19-* cells (Fig. 4A,B). On the other hand, cell cycle analysis showed an arrest in the G2/M phase (*p* < 0.0001), a characteristic of increased mitotic activity in proliferative cells (Fig. 4C). H19 downregulation, accelerated cell growth and cell cycle arrest in the G2/M phase observed in the C2C12 *H19*- cells. This increased proliferative activity seems to facilitate chromosomal rearrangements in the edited cells since the number of normal metaphases was decreased in the C2C12 *H19*- cells (43%) when compared to the non-edited cells (67%) (*p* = 0.0231) (Fig. 5C). Nevertheless, the C2C12 *H19-* knockdown cells presented a higher number of translocation events, including the presence of four translocations that was not observed in the non-edited cells (Fig. 5A,B, Supplementary Figure S2).

## Discussion

CRISPR-mediated *H19* knockdown in C2C12 myoblast cell lineage promotes reduced gene expression and altered cell fate, such as increased cell growth, altered cell size and internal complexity with G2/M cell-cycle arrest and increased chromosomal abnormalities. The imprinted *H19* lncRNA is a maternally expressed gene essential for cell differentiation and normal development, with intense transcriptional activity during embryogenesis which decreases after birth^11^. In normal conditions, the *H19* is a tumor suppressor gene^8^ and due to its role in growth-related pathways and differentiation, alterations in *H19* gene expression are related to the carcinogenic process^16^. However, its role in cancer initiation, progression and metastasis remain controversial and the lncRNA *H19* may act as an oncogene during tumorigenesis, being one of the key genes in cancer development^5^.

Gene edition strategies allow functional studies of important molecular markers and the pathways related to the tumorigenesis process. To observe the role of lncRNA *H19* in cancer initiation we sought to develop a non-cancer knockdown murine cell line. The edition strategy resulted in small deletion near the first gRNA1 (146 bp) without the removal of the whole promoter, suggesting that the gRNA2 was not able to guide the edition in the selected region. Although plasmid concentrations were similar for gRNA1 and gRNA2, other experimental details as the transfection efficiency may change the average performance of both guides in the edited clonal cell lines^17^. In addition, the existence of two products with different sizes and similar intensity may indicate that only one allele was edited, resulting in a monoallelic modification^17^, that was responsible for the downregulation of gene expression. Since the *H19* is an imprinted gene with monoallelic expression, probably the unmethylated maternal allele was edited and the methylation at the H19DMR in the paternal allele neighboring the *H19* promoter and the conformational changes in the promoter region due to methylation at the H19DMR may have prevented the edition.

Despite the small promoter deletion, the *H19* gene expression was decreased in the edited C2C12 cells. It was observed that the removed region presented four TFBS with the alpha and beta glucocorticoid receptor family and estrogen receptor. These receptors act as transcription factors and their binding sites are widely present in promoter and enhancer regions, regulating the expression of many genes in the mammalian genome, such as for the inhibition of pro-inflammatory signaling pathways like *ANXA1, NFKBIA, DUSP1, GILZ* and *ZFP36*^18,19^. The gene activation by those transcription factors is associated with the antiproliferative effect and cell cycle arrest in the G1 phase in mammary epithelial, fibroblasts and hepatoma cells^20^.

We observed that *H19* downregulation in the non-cancer cell line promotes cell growth and morphological alterations, like increased cell volume and intracellular granularity (Supplementary Figure S1) and cell cycle arrest in the G2/M phase, without changes in viability. These biological alterations, as sustaining proliferative signaling and evading growth suppressors, are the main characteristics observed in cancer cells^21^. Since the *H19* is a known oncofetal gene, its role in carcinogenesis has been widely studied. Decreased lncRNA *H19* expression resulted in cell proliferation and migration in papillary thyroid carcinoma cell line and may contribute to lymph node metastasis^22^. Yu et al. (2013)^23^ observed that the reduction of *H19* expression reduced cell growth in a human choriocarcinoma cell line. *H19* knockdown also showed antiproliferative effects, reduced invasion and migration and induced apoptosis in the Y79 retinoblastoma cell line^24^. In accordance, downregulation of *H19* in SW579 and TPC-1 thyroid cancer cell lines decreased cell viability, migration and invasion^25^.

Yoshimizu and collaborators (2008)^8^ showed that *H19*-null murine embryos presented increased weight and size of tumors after induction of teratocarcinoma, even in those animals’ knockout for *IGF2* gene (*IGF2*-/-). After induction of colorectal cancer, the *H19* knockout model presented twice more polyps, suggesting that the *H19* may play a role in the initiation and progression of tumorigenesis acting as a tumor suppressor gene. In contrast, a xenografic model of glioblastoma showed that *H19* is related to tumorigenicity and stemness with an important role in cancer development as an oncogenic marker^26^. Considering human studies, Sun and collaborators (2017) reported increased expression of *H19* in multiple myeloma bone marrow that was correlated with a lower survival rate^27^. Furthermore, *H19* may be a molecular marker of prognosis which was associated with malignancy in breast tumors^28^ and metastasis in pancreatic cancer^29^.

Additionally, the *H19* knockdown C2C12 cell line presented increased chromosomal alterations, being more susceptible to events of two, three and four translocations than the non-edited cells. Disruption in the cell cycle and uncontrolled proliferative activity increases the risk of aberrant DNA synthesis and genomic instability leading to random mutations and chromosomal rearrangements^21^. Moreover, the CRISPR/Cas9 edition may generate genomic instability and chromosomal alterations due to unpredicted off-targets^30^, especially in aneuploidy cells as those used in in vitro studies^30–32^. The G2/M cell-cycle arrest corroborates with increased translocation events, in which the checkpoint acts in response to DNA damage and the repair might occur^33^. However, neither the reduced *H19* gene expression nor the cellular and chromosomal alterations observed had implications on natural morphological characteristics of myoblast cells, as the smooth-surfaced ovals nuclei and elongated cytoplasm patterns^34^.

Although the precautions in the selection of gRNAs with high performance and reduced off-target effects in silico, the edition result showed a modification smaller than expected. The accuracy of modifications via CRISPR/Cas9 has been previously reported, as well as the asymmetry between the activity of gRNA libraries^35,36^, unexpected deletions and complex rearrangements (insertions and inversions)^30,37^. Although off-targets were predicted in silico using the CRISPR design tool, one limitation of this study is that we did not perform whole-genome sequencing since this is a high-cost analysis that requires a detailed bioinformatics investigation. However, CRISPR design and all experiments were carefully conducted following all guidelines to minimize the off-target effects. As previously reported by Zang and collaborators (2015)^38^, the Cas9 activity is tightly controlled by the gRNA and PAM (NGG) sequences. The strength of base pairing in the “seed” sequence of the gRNA determines Cas9 specificity, mainly from the first to five base pairs of guide region adjacent to PAM, which is considered the “true seed region”, a critical factor for DNA targeting^39^.

Less than 1% of the “seed + NGG” sites in the genome are bound to Cas9, and most of them are in promoters, enhancers, and gene bodies^14^. According to our *in-silico* analysis, the seed sequence of the predicted off-targets does not present full complementarity to any promoter or gene body sequence, which increases on-target cleavage efficiency (Supplementary Table S1). Indeed, the chosen gRNA are genetically distant from the off target sequences, since the greater the distance the smaller is the non-specific pairing. Other experimental conditions were carefully designed to avoid these effects, as the concentration of Cas9 and gRNA^14,39^.

The permanent effects of the CRISPR/Cas9 gene edition are not completely understood and the main concern is the unpredicted changes in the genome or an undesired immune response^40^. Nevertheless, CRISPR is an important and powerful tool in genome-editing technology, with a permanent edition that allows functional studies and the development of genetically modified organisms with potential use in the genetic correction of disease mutations. Regardless of the technical disadvantages, CRISPR-mediated deletion in the *H19* promoter region decreased gene expression and resulted in increased proliferative activity, cell volume and intracellular granularity, without changes in viability characteristics frequently observed in the cancer initiation process. Nevertheless, this increased proliferative activity may facilitate chromosomal rearrangements.

Carcinogenesis is a multistep process, that includes cancer initiation, progression, and metastasis, with a complex and multifactorial network of genetic and epigenetic modifications cell/tissue and time-specific^32^. The controversial role of lncRNA *H19* as a tumor suppressor or oncogenic activity seems to be depending on the stage of the tumorigenic process, microenvironment context and the studied object, such as in vitro, in vivo and human samples^5^. Therefore, alterations in the imprinted lncRNA *H19*, essential for differentiation and growth-related pathways, may result in different effects in cancer cells. The C2C12 *H19-* cell lineage showed alterations similar to cancer cells, suggesting an important role of *H19* in the cancer initiation process as a tumor suppressor gene. To our knowledge, this is the first report of a permanent genetic edition of the *H19* gene in a non-tumor cell line. The development of genetically modified lineages allows functional and biological studies of important molecular markers involved in tumorigenesis, and the C2C12 *H19-* knockdown cell line may be an important model to elucidate the role of the *H19* gene in tumorigenesis, prognosis, metastases, as well as drug resistance and targeted therapy.

## Materials and methods

### Cell culture

Myoblasts C2C12 cells (ATCC CRL-1772) were thawed at passage 5 and cultured in Dulbecco’s modified Eagle’s medium (DMEM; #21969; Gibco) supplemented with 10% fetal bovine serum (FBS) and 100 U mL^−1^ of penicillin in a humidified 10% CO_2_ atmosphere at 37 °C. Cells were grown in T75 flasks and 96-well plates (Thermo Fisher Scientific, USA). The medium was replaced every 3 days and cells were sub-cultured twice per week using 0.25% (w/v) Trypsin–0.53 mM EDTA solution. Cell growth was monitored using the Primovert inverted microscope (Carl Zeiss).

### Construction of DNA vectors

The guide RNAs (gRNAs) were designed using the *CRISPR design* platform from Massachusetts Institute of Technology (http://crispr.mit.edu) and two gRNA were selected to delete 1485 bp of the promoter region predicted by the Eukaryotic Promoter Database^41^. Potential off-targets were predicted using the same guide design platform, assuming that the best gRNAs would be those with the lowest off-target events in promoters and exons (Supplementary Table S1). To observe the specificity of our gRNAs a nucleotide BLAST alignment was performed (https://blast.ncbi.nlm.nih.gov) (Supplementary Table S2). The following gRNA1 5′-CATCAGCAGACTAAAGGCCG-3′ and gRNA2 5′-CGTGGCGGCTGGTCGGATAA-3′ were obtained by synthesis (Sigma, San Luis, EUA) adding the *Bbs*I restriction site at their 5′ ends. The CRISPR-deletion strategy was based on double-strand breaks using two independent vectors containing each gRNA ligated into the pX458 plasmid (Addgene #48138) at the *Bbs*I restriction site. The pX458 vector also contains a green fluorescent protein (GFP) and an ampicillin resistance gene. The gRNA is located after the U6 promoter.

### CRISPR/Cas9 H19 knockdown in C2C12 cells

Transfections were conducted by electroporation using the Neon Transfection System (Thermo Fisher Scientific). Cells were grown to the density of 5 × 10^6^ and submitted to electroporation with 7.5 μg of each CRISPR/Cas9 vector with a pulse of 1650 V for 10 mS. After electroporation, cells were cultured in DMEM media supplemented with 10% fetal bovine serum (FBS) without antibiotics for 24 h at 37 °C and 10% CO_2_ atmosphere. The transfection was confirmed by the detection of GFP through fluorescence microscopy (Eclipse Ti-S®, Nikon). The isolation of single-cell clones was performed using limiting dilution in 96-well plates. After 24 h of transfection, the viability of the cells from the transfected pool was verified using trypan blue solution (10%). The selection of clones was done by plating them at a very low density, around 0.5 cells per well, to ensure that each well would have no cell or at least one cell.

### Sanger sequencing

To confirm the edition at *H19* promoter, the cultured cells were trypsinized and washed to remove the remaining culture media and resuspended in PBR 1X. Subsequently, genomic DNA was obtained using the MasterPure™ Complete DNA and RNA Purification Kit (Illumina, San Diego, EUA) according to the manufacturer's instructions. DNA concentration was determined using a Nanodrop 2000c spectrophotometer (Thermo Fisher Scientific). To confirm the edition, we performed PCR amplification using *Pfu DNA polymerase* (Promega) and the following primers flanking the region of interest: forward 5′-GGGGGATATAGCAGGGGTGT-3′ and reverse 5′-GCTATACCTTCACTGCCCAGGT-3′. The thermal cycling conditions were: 95 °C for 5 min; followed by 30 cycles of 94 °C for 30 s, 62 °C for 30 s, and 72 °C for 2 min; and a final extension of 72 °C for 5 min. The PCR product was purified from 1% agarose gels using the QIAquick PCR Purification kit (Qiagen) and both reverse and forward primers were used for Sanger sequencing using *BigDye™ Terminator v3.1 Cycle Sequencing Kit* (Thermo Fisher Scientific, Waltham, EUA) in the instrument ABI 3100 Automated DNA Sequencer (Thermo Fisher Scientific, Waltham, EUA).

### Bioinformatics analysis of transcription factor binding sites

The prediction of transcription factor binding sites in the C2C12 Wt and C2C12 *H19-* promoter sequence was performed with the PROMO software version 3.0.2 database using the TRANSFAC version 8.3, with a cut‐off for the dissimilarity matrix at 1% and 99% similarity (http://alggen.lsi.upc.es/cgi-bin/promo_v3/promo/promoinit.cgi?dirDB=TF_8.3). PROMO bioinformatics program was used for predicting potential motif binding sites for transcription factors absent because of the edition^42^.

### Gene expression analysis

Gene expression was evaluated using reverse transcription associated with quantitative real time PCR (RT-qPCR). Total RNA from both isogenic edited and unedited colonies were isolated using MasterPure ™ Complete DNA and RNA Purification Kit (Illumina). DNA quality and concentration were determined using a Nanodrop 2000c spectrophotometer (Thermo Fisher Scientific). Complementary DNA (cDNA) synthesis was performed using the *SuperScript IV Reverse Transcriptase* (Thermo Fisher Scientific) with Oligo d(t)_20_ primers, according to the manufacturer’s instructions. The following primers were used to amplify the *H19:* forward 5′-CGACGGAGCAGTGATCGG-3′ and reverse 5′-GACAGGTGTGGTCAATGTGA-3′ and the reference gene, beta actin (*Actb*): forward 5′-CCTGAACCCTAAGGCCAACC-3′ and reverse 5′-TGGATGGCTACGTACATGGC-3′. Each 10 µL reaction solution contained: 1X of *SYBR® Premix Ex Taq II*, 0.2 µM of each primer, 2 µL (100 ng) of cDNA and RNAse free water. Each reaction was carried out in triplicate in three independent experiments. The thermal cycling conditions were 95 °C for 3 min (1 cycle); followed by 40 cycles of 95 °C for 15 s and 59 °C for 1 min. A melting curve analysis was added (95 °C for 15 s, 65 °C for 15 s, 95 °C for continuous acquisition) to demonstrate the specificity of the qPCR products, as revealed by a single peak. The 2^−∆∆Cq^ method was used to calculate the relative gene expression levels^43^.

### Cell proliferation assay

Cells were seeded into 96-well culture plates at a density of 6000 cells/well for both groups on day zero (D0) and the cell growth was monitored for six days. C2C12 Wt and C2C12 *H19-* were cultured as previously described. Cellular viability was identified by the MTT assay. Briefly, MTT (20 μl, 5 mg/mL) was added to each well, followed by incubation for 4 h at 37 °C, 5% CO_2_. After the incubation time, plates were centrifuged, the supernatant aspirated and the MTT formazan product was dissolved in DMSO (200 µL). The content of the plates was mixed for 20 min, and the absorbance was measured using a multiplate reader (DTX 880 Multimode Detector, Beckman Coulter, Inc. Fullerton, California, EUA) at 525 nm.

### Cell morphology

The cell morphology was evaluated after seeding 40,000 cells/well in a 24-well plate over a round glass coverslip. After 24 h, the round glass coverslip was removed from the plates, fixed with methanol and stained with a quick panoptic kit (Laborclin, Brazil). The evaluation of cellular morphological changes was performed by light microscopy (Olympus, Tokyo, Japan). The images were chosen to represent the general picture prevalent in all coverslips for a given group (C2C12 Wt or C2C12 *H19-*).

### Flow cytometry

Cell morphology, granularity and membrane integrity were evaluated by propidium iodide (5 µg/mL, Sigma Aldrich Co., St. Louis, MO, USA) exclusion using flow cytometry. Briefly, 24 h after plating, C2C12 Wt and C2C12 *H19-* cells were harvested and incubated for 10 min with propidium iodide, in the dark. After the incubation time, fluorescence was measured by flow cytometry in a *Guava EasyCyte Mine* in three independent experiments. For cell cycle analyses, cells were harvested and incubated in the dark, at room temperature, with a solution containing RNAse A (50 µg/mL), propidium iodide (5 µg/mL), 0.1% sodium citrate and 0.1% Triton X-100 for 40 min. Data were analyzed by ModFit LT software (Verity Software House, Inc., Topsham, ME).

### Karyotype

Both C2C12 Wt and C2C12 *H19-* were treated with 50 ng/mL Colcemid^®^ for 1 h. Following Colcemid^®^ treatment, the cell culture flasks were incubated with 1 mL of a 0.05% Trypsin–EDTA solution at 37 °C and 5% CO_2_. Trypsinization was stopped after 5 min with the addition of attachment medium, and the cells were gently collected in 15 mL tubes and pelleted at 1.200 rpm for 10 min. The cell pellet was gently resuspended in 75 mM KCl. After 15 min of incubation, 0.5 ml of fixative (3:1 methanol to acetic acid—Carnoy's solution) were added to each tube to stop hypotonic solution activity and the tubes were immediately centrifuged for 10 min at 1.200 rpm. The supernatant was discarded, and 10 mL of cold Carnoy's solution was added to each tube. This step was repeated two more times. For this analysis, the fixed cells were dropped onto slides and digested with Trypsin (1:250) for three seconds before Wright staining. Metaphases were analyzed after G-banding and the evaluation of chromosomal abnormalities in booth edited and non-edited cells were blinded without previous knowledge of which samples had a deletion of *H19*, to reduce or eliminate the experimenter's bias.

### Statistical analysis

Data were presented as mean ± standard error of the mean (SEM) from independent experiments performed in at least triplicates with unpaired Student's t-test comparisons. The chi-square statistic test was used to perform the karyotype analysis between the edited and non-edited cells. All statistical analyses and graphs were carried out using GraphPad Prism 8.0 (Intuitive Software for Science, San Diego, California, USA). *p* < 0.05 was considered statistically significant.

## Supplementary Information

Supplementary Information.
