# Supplementary material for: CRISPR/Cas9 small promoter deletion in H19 lncRNA is associated with altered cell morphology and proliferation
Source: Sci Rep. 2021 Sep 15;11:18380. doi: 10.1038/s41598-021-97058-0 (PMC8443613; doi:10.1038/s41598-021-97058-0)
Supplement: Supplementary file 1 — Supplementary Information. [file 41598_2021_97058_MOESM1_ESM.docx]

**SUPPLEMENTARY MATERIAL**

**CRISPR/Cas9 Small Promoter Deletion in *H19* lncRNA is associated with altered cell proliferation and chromosomal abnormalities**

**CRISPR/Cas9-mediated small promoter deletion of the *H19* lncRNA is associated with altered cell morphology and increased proliferation**

Renan da Silva Santos^1^, Daniel Pascoalino Pinheiro^1^, Louhanna Pinheiro Rodrigues Teixeira^2^, Sarah Leyenne Alves Sales^1^, Maria Claudia dos Santos Luciano^1^, Mayara Magna de Lima Melo^3^, Ronald Feitosa Pinheiro^3^, Kaio César Simiano Tavares^2^, Gilvan Pessoa Furtado^4^, Claudia

**Supplementary Figure S1.** Flow cytometry histograms. The analysis of cell size (A) and granularity (B) are represented on the X axis by Forward Scatter and Side Scatter, respectively.


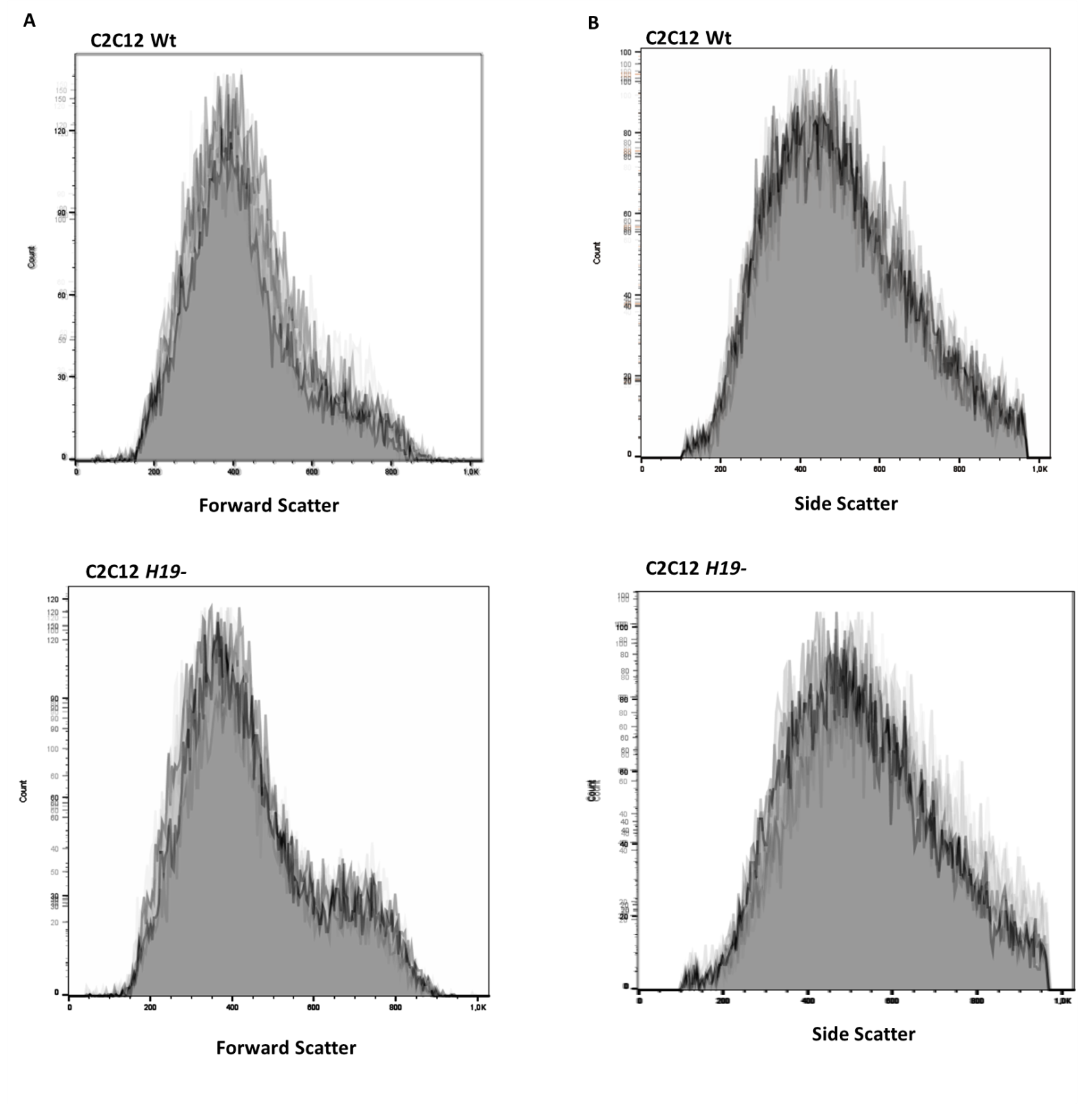


**Supplementary Figure S2**. Normal metaphase and translocation events in the C2C12 *H19-* and C2C12 Wt.


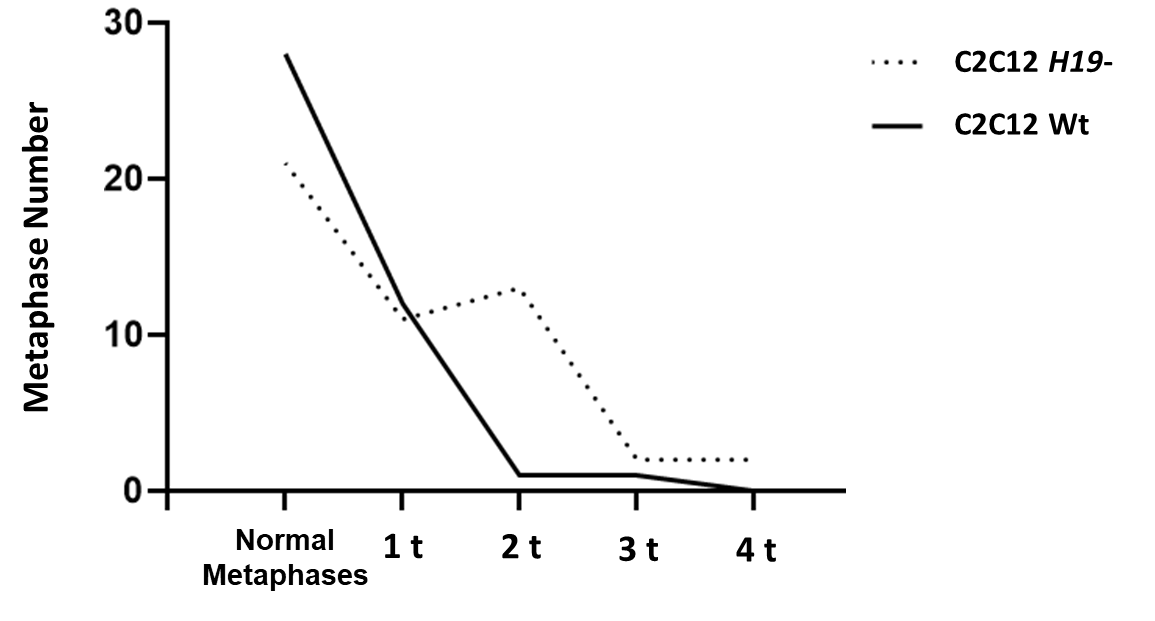


**Supplementary Table S1.** Characteristics of potential off-target events in exons.

| Target | Gene* | Position | Function* | Muscle  Expression | | Sequence | Mismatch Number | Mismatch in Seed | |
| --- | --- | --- | --- | --- | --- | --- | --- | --- | --- |
| ON | gRNA 1 | chr7:142579385-  142579404 | | | CATCAGCAGACTAAAGGCCG | | | | |
| OFF 1 | *Kmt2a* | chr9:44843351-44843373:- | lysine (K)-specific methyltransferase 2A | mild | CA**GA**AGCAGACTAAAGG**TA**G | | 4 | 2 | |
| OFF 2 | *Ppard* | *chr17:28300200-28300222:+* | peroxisome proliferator activator receptor delta | mild | CA**C**CAGCAG**C**CTAAA**A**GC**AG** | | 4 | 2 | |
| OFF 3 | *Lrig1* | *chr6:94626400-94626422:-* | leucine-rich repeats and immunoglobulin-like domains 1 | mild | CATCAGCAG**G**CT**G**A**C**GG**A**CG | | 4 | 3 | |
| OFF 4 | *Thsd1* | *chr8:22238502-22238524:+* | thrombospondin, type I, domain 1 | mild | CAT**G**AG**G**AGACTA**C**AGGCC**T** | | 4 | 2 | |
| OFF 4 | *4930412O13Rik* | *chr2:9881899-9881921:-* | RIKEN cDNA 4930412O13 gene | absent | CA**G**CAGCAG**C**CT**C**AAGGCC**T** | | 4 | 2 | |
| ON | gRNA 2 | chr7:142577859-  142577878 | | | CGTGGCGGCTGGTCGGATAA | | | | |
| OFF 1 | *Azi2* | *chr9:118062058-118062080:-* | 5-azacytidine induced gene 2 | mild | **A**GTGGCGGC**A**GGT**T**GGATAA | | 3 | | 1 |
| OFF 2 | *Dnm2* | *chr9:21506365-21506387:-* | dynamin 2 | mild | CG**GCT**CGGCTGG**G**CGGATAA | | 4 | | 1 |

*Nomenclature and function according to Genbank (https://www.ncbi.nlm.nih.gov/genbank/). Mismatches are marked in bold. Seed sequence is underlined.

**Supplementary Table S2.** Top 10 BLAST alignment sites for guides RNA.

| gRNA 1 | Query  Cover | E-value | Accession  Number |
| --- | --- | --- | --- |
| [Mus musculus H19 and muscle-specific Nctc1 genes, complete sequence](https://blast.ncbi.nlm.nih.gov/Blast.cgi#alnHdr_2970434) | 100% | 0.014 | AF049091.1 |
| Mus musculus chromosome 7, clone RP23-209O22, complete sequence | 100% | 0.014 | AC013548.13 |
| Mus musculus genomic DNA, chromosome 7 clone:B229F13, complete sequences | 100% | 0.014 | AP003183.2 |
| Mus musculus (H19) gene, 5' non-transcribed region sequence | 100% | 0.014 | U19619.1 |
| Mus musculus BAC clone RP24-548E5 from chromosome 1, complete sequence | 80% | 3.4 | AC125310.5 |
| Mouse DNA sequence from clone RP23-139F8 on chromosome 2, complete sequence | 80% | 3.4 | AL732317.13 |
| Mus musculus hydroxyacyl-CoA dehydrogenase trifunctional multienzyme complex subunit alpha (Hadha) | 75% | 13 | NM_178878.3 |
| Mus musculus VISTA enhancer mm1322 (LOC109280138) on chromosome 18 | 75% | 13 | NG_054407.1 |
| Mus musculus targeted non-conditional, lacZ-tagged mutant allele Mapre1:tm1e(EUCOMM)Hmgu; transgenic | 75% | 13 | JN964650.1 |
| Mus musculus targeted KO-first, conditional ready, lacZ-tagged mutant allele Mapre1:tm1a(EUCOMM)Hmgu; transgenic | 75% | 13 | JN964401.1 |
| gRNA 2 | **Query**  **Cover** | **E-value** | **Accession** |
| Mus musculus H19, imprinted maternally expressed transcript (H19), transcript variant 2, long non-coding RNA | 100% | 0.014 | NR_130974.1 |
| Mus musculus H19, imprinted maternally expressed transcript (H19), transcript variant 1, long non-coding RNA | 100% | 0.014 | NR_130973.1 |
| Mus musculus 5 days embryo whole body cDNA, RIKEN full-length enriched library, clone:I0C0030C13 product:Mus Musculus h19 fetal liver mRNA (H19), mRNA, full insert sequence | 100% | 0.014 | AK145379.1 |
| Mus musculus 12 days embryo male wolffian duct includes surrounding region cDNA, RIKEN full-length enriched, clone: 6720441I24 product:Mus Musculus h19 fetal liver mRNA (H19), mRNA, full | 100% | 0.014 | AK135234.1 |
| Mus musculus H19 and muscle-specific Nctc1 genes, complete sequence | 100% | 0.014 | AF049091.1 |
| Mus musculus chromosome 7, clone RP23-209O22, complete sequence | 100% | 0.014 | AC013548.13 |
| Mus musculus genomic DNA, chromosome 7 clone:B229F13, complete sequences | 100% | 0.014 | AP003183.2 |
| Mouse H19 gene | 100% | 0.014 | X07201.1 |
| Select seq XM_030245069.1 PREDICTED: Mus musculus cadherin 23 (otocadherin) (Cdh23), transcript variant X1, mRNA | 70% | 53 | XM_030245069.1 |
| Select seq NM_001369056.1 Mus musculus SECIS binding protein 2-like (Secisbp2l), mRNA | 70% | 53 | NM_001369056.1 |

The highlighted lines represent alignments with higher Query Cover and E-value.
